# Validity and Reliability of the Computrainer Lab™ During Simulated 40 and 100 km Time-Trials

**DOI:** 10.3389/fspor.2021.735046

**Published:** 2021-09-01

**Authors:** David Jeker, Jonathan Gosselin, Jean-Marc Drouet, Eric D. B. Goulet

**Affiliations:** ^1^Department of Kinanthropology, Faculty of Physical Activity Sciences, University of Sherbrooke, Sherbrooke, QC, Canada; ^2^VÉLUS Laboratory, Department of Mechanical Engineering, University of Sherbrooke, Sherbrooke, QC, Canada; ^3^Research Centre on Aging, University of Sherbrooke, Sherbrooke, QC, Canada

**Keywords:** cycling, cycling ergometer, performance, validity, assessment

## Abstract

The validity and reliability of the Computrainer Lab^™^ (CT) was assessed, for the first time, using a high-precision motor-driven calibration rig during simulated variable intensity 40 and 100 km time-trials (TTs). The load patterns imposed by the CT were designed from previously published studies in trained cyclists and included multiple 1 or 4 km bursts in power output. For the 40 and 100 km TTs, cluster-based analyses revealed a mean measurement error from the true workload of respectively 0.7 and 0.9%. However, measurement errors were dependent upon the workload variations, fluctuating from 0.2 to 5.1%. Average biases between repeated trials were contained within ± 1.1% for both TTs. In conclusion, using 40 and 100 km TTs containing 1 or 4 km bursts in power output, the present results indicate that (1) the CT can reliably be used by scientists to determine differences between research interventions; (2) the CT provides valid results of power output when data are being analyzed as a whole to derive one mean value of power output and; (3) variations in workload make it difficult to determine at any one time the veracity of the true power output produced by the athlete.

## Introduction

Cycling time-trials (TTs) are being used under controlled research settings to assess the effect of an intervention or condition on endurance performance. Although debatable (Amann et al., 2008), they have been demonstrated to be more reliable than either fixed-intensity (Jeukendrup et al., 1996) or incremental cycling tests to exhaustion (Coakley and Passfield, 2018). Unarguably, however, their ecological validity is greater than cycling tests during which workloads are being dictated, making them a premier choice for scientists aiming to optimize the external validity of their research findings. Cycling TTs can easily be performed in a controlled research environment using a cycling ergometer, although their ecological validity will be substantially enhanced if research participants use their own bike on a resistance trainer.

The Computrainer^™^ (CT, RacerMate, Seattle, WA, USA) is an electromagnetically braked resistance trainer applying friction to the rear wheel of a standard bike, which can be used in a cadence dependent and independent mode. It has been used for research purposes to measure cycling TT performances (van Essen and Gibala, 2006; Berardi et al., 2008; Cermak et al., 2012; Wilkerson et al., 2012; Dyer and McKune, 2013; Lamberts, 2014; Nieman et al., 2015; Peveler et al., 2017; Adams et al., 2019; Ely et al., 2019; Perreault-Briere et al., 2019), TT's reliability (Lamberts et al., 2009; Sparks et al., 2016) or analyze pacing strategies during TTs (Atkinson and Brunskill, 2000; Micklewright et al., 2010; Jones et al., 2015; Jones and Williams, 2017; Whitehead et al., 2018). Although the company stopped producing the CT in 2017, this resistance trainer is still widely used (Dionne et al., 2018; Nieman et al., 2018a,b; Rønnestad et al., 2018; Silva-Cavalcante et al., 2018, 2019; Ely et al., 2019; Evens and Danoff, 2019; Chidnok et al., 2020; Haugen et al., 2020) and will likely be used for several more years by research teams all around the world, included ours (Jeker et al., 2020; Claveau et al., 2021), for several reasons. First, it is a plugged-in trainer, which confers more confidence and assurance to the researcher that a connectivity problem should not be occurring during an experiment; with the use of a wireless trainer, the slightest loss of signal could result in a failed experiment. Second, it provides the advantage for the researcher to use the bike brought by the participant as is, without the need for rear-wheel removal and a cassette change, which is the case for the more modern direct-drive trainer. Third, the CT has acquired over the years a strong reputation among laboratories and researchers for its ruggedness and durability. Finally, the company has established a replacement parts program, which will enable researchers to keep using the CT for a long time, if it is their wish.

Knowing the reliability and validity of performance-measurement devices is critical. With regards to resistance trainers, they may help researchers (1) determine the smallest absolute worthwhile change in performance that could be detected from one trial to the other, (2) compute and determine an optimal sample size, (3) improve the ability to interpret changes in performance, (4) determine whether the enhancement in performance can be useful for out-of-doors exercise conditions or provide assurance that it could be reproduced under those conditions and; (5) establish the credibility of findings observed in previous and future studies.

The validity of the CT has already been assessed for fixed workload exercises, i.e., in a cadence independent mode, using a torque reaction calibration rig enabling direct measurement of the true workload produced by the CT (Drouet et al., 2008). Using a device instead of humans to determine the validity or reliability of resistance trainers offers the premier advantage of removing variability associated with biological variations and sampling error, thereby enabling determining true validity or reliability. It was previously shown that at a pedaling cadence ranging from 80 to 100 revolutions per min (RPM), the workloads produced by the CT were ~10 to 25 W lower than those generated by the calibration rig from 100 to 400 W (Drouet et al., 2008). Moreover, it was observed that, at a pedaling cadence ranging from 80 to 100 RPM, the CT generated lower (~20 to 50 W) workloads than those from the calibration rig between 100 to 350 W (Guiraud et al., 2010). Altogether, findings from those studies indicate that the validity of the CT is questionable when it is being used under a cadence independent mode.

To our knowledge, the validity and reliability of the CT have never been assessed under TT conditions, i.e., in a cadence dependent mode. Therefore, the goal of this study was to determine the validity and reliability of the CT during 40 km and 100 km TTs using a motor-driven calibration rig. The results of this study will be relevant for future research using the CT, but may also clarify previous results obtained with it.

## Methods

### Overview of the Experiment

**Figure 1:**
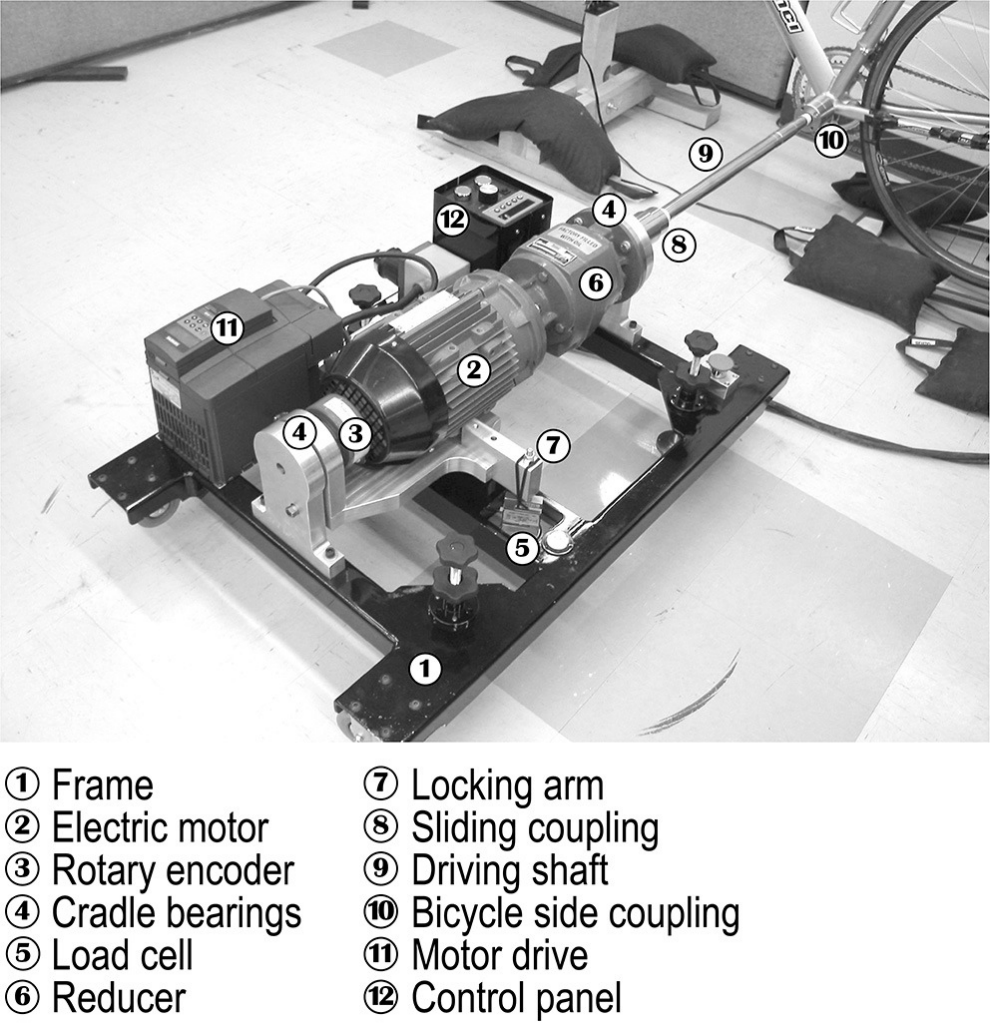
Experimental setup.

Two 40 and 100km TTs were conducted on a virtual flat course with a road bike mounted on a CT Lab^™^. The bike was powered by a motor-driven, high-precision calibration rig (Figure 1). The 40 km TTs were interspersed by 31 days, whereas the 100 km TTs by 28 days; these delays occurred because of the lack of availability of the calibration rig. All experiments were conducted in the same laboratory, at standardized temperatures (20–22°C) and humidity levels (20–40%). The TTs were conducted at an average workload that could be sustained by endurance-trained competitive elite cyclists or triathletes during distances of 40 (Coyle et al., 1991) and 100 km (Schabort et al., 1998).

### Road Bike and Drivetrain Components

A standard aluminum road bicycle (Radon C2, Argon 18, Montreal, Canada) was mounted onto the CT. The bicycle was equipped with a Shimano (Sakai, Japan) 105 R5600 rear derailleur, a Shimano BB-5500 Octalink bottom bracket, a Shimano CN-HG 53, 9-speed chain, a Shimano CS-HG 70, 9-speed cassette (12–23 teeth), a 53 x 42-tooth SRM crankset (Jülich, Germany) and a Mavic 32-spoke Open Pro 700 C rear-wheel (Annecy, France). Prior to starting the experiment, the chain was lubricated with a wax-based lubricant (Clean Ride, White Lightning Co., Hauppauge, NY, USA) and a Tacx 700 x 23 C trainer-specific tire (T1390, Wassenaar, Netherlands) was mounted onto the rear rim. The tire was inflated to 110 psi (7.6 bars) before each test. The same tire was used for all experiments.

### Calibration Rig

The bike was powered by a speed-controlled motor which provided the required torque to drive the left side of the bottom-bracket axle via a driving shaft at a pre-selected rotating angular velocity that was independent from the workload imposed by the CT. The setup requires removal of the left-hand side crank arm. The power transferred to the bicycle is linearly related to the torque and angular velocity. The torque depends upon the force measured by a load cell and the constant lever arm length corresponding to the distance between the rotating shaft and the load cell. The calibration rig can be operated at pre-selected pedaling cadences ranging from 80–130 RPM, by 10 RPM increments which affect the angular velocity. Including all sources of uncertainty, the measurement error of the calibration rig for a nominal power level ranging from 50 to 600 W is ± 0.9% (Drouet et al., 2008).

### Production and Measurement of Workload

Comparisons were made by contrasting, in real-time, the workloads delivered by the calibration rig to those measured and displayed by the CT. Prior to performing the tests, the CT was calibrated based on the manufacturer's recommendations for a flat course after a 10 min fixed-intensity warm-up at 350 W, with the aim of achieving a press-on force of 8.9 ± 0.2 N. As an increase in tire temperature caused by its friction on the trainer's shaft may lead to a reduction in rolling resistance and affect the accuracy of the CT (Evens and Danoff, 2019), the warm-up intensity was chosen to be as close to the TTs intensity. Because of the difficulty associated with gear shifting while the calibration rig is being operated, the bicycle was always used in the same gears, that is on the 53 x 15T, i.e., one complete rotation of the crank caused the rear wheel to rotate 3.5 times. Therefore, variations in workloads were achieved by modifying the RPM. Two different cadences (90 and 100 RPM) were used for the 40 km TTs and three (80, 90 and 100 RPM) for the 100 km TTs. The workload produced by the CT was computed and recorded with the RacerMate One^™^ software (RacerMate, Seattle, WA, USA). Workload values produced by both the CT and the calibration rig were recorded every 500 m.

### Statistical Analysis

The presence of heteroscedasticity was verified for each segment of the 40 and 100 km TTs by correlating the measurement errors with the distance covered, using either simple or polynomial linear regression models that best fitted the line of data, based on the highest-associated R^2^ value. Validity and reliability data were determined using the measurement errors (calibration rig—CT), coefficients of variation (CV) and 95% standard errors of the estimate (SEE), when heteroscedasticity was present, or standard errors of the measurement (SEM), when it was not. Statistical analyses were performed using the R software version 3.6.3 (R foundation for Statistical Computing, Vienna, Austria).

## Results

### Completion Times and Workloads

Whether the TT was 40 or 100 km long, 6 s separated the first from the second trial, as measured by the Racermate One^™^ software with completion times of respectively 57 min 28 s and 57 min 22 s for the 40 km TTs, compared to respectively 2 h 37 min 49 s and 2 h 37 min 43 s for the 100 km TTs. The mean workloads produced by the calibration rig and CT during the 40 km TTs were of respectively 362 vs. 359 W (Δ of −3 W) for the first trial, compared to 363 vs. 360 W (Δ of −3 W) for the second trial. In comparison, the mean workloads produced by the calibration rig and CT during the 100 km TTs were of respectively 286 vs. 282 W (Δ of −4 W) for the first trial, compared to 284 vs. 282 W (Δ of −2 W) for the second trial. Data for the 40 km TT were congruent in that the completion time was lower for trial 2 than trial 1, the workloads measured by the CT and calibration rig were higher for trial 2 than trial 1 and similar differences in workload were observed between the CT and calibration rig. However, this was not the case for the 100 km TT where completion time was lower for trial 2 than trial 1, yet the workloads produced and measured by the CT were similar between trials but those measured by the calibration rig were different, with a greater workload required to meet that of the CT during the first than the second trial. Nevertheless, the difference between these workloads was just outside the ± 0.9% power uncertainty of the calibration rig with a difference of 0.98%.

### Validity

**Figure 2:**
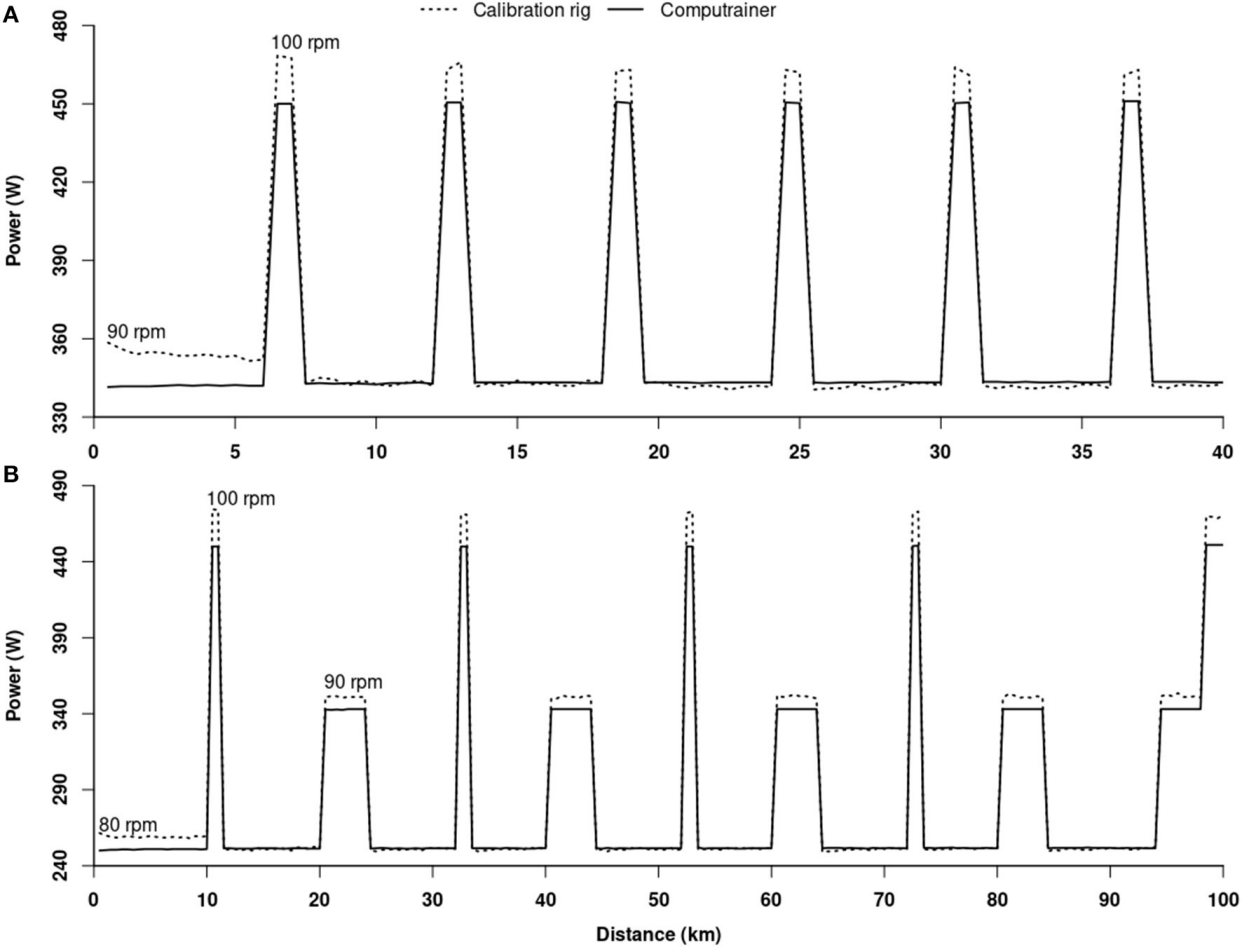
Changes in workloads recorded by the CT and calibration rig during the 40 **(A)** and 100 **(B)** km time-trials.

Figure 2 shows the changes in workloads produced by the calibration rig and CT throughout the 40 (A) and 100 (B) km TTs. A pattern emerges showing that prior to the first change in cadence, the workloads produced by the calibration rig were higher than those of the CT, independent of the TT distance. Then, the workloads produced by both the calibration rig and CT were similar at the lowest cadences for both TT distances, and again disproportionate for each of the 40 and 100 km TTs at the highest or intermediate (100 km TT) cadences, with the calibration rig producing higher workloads than the CT.

**Figure 3:**
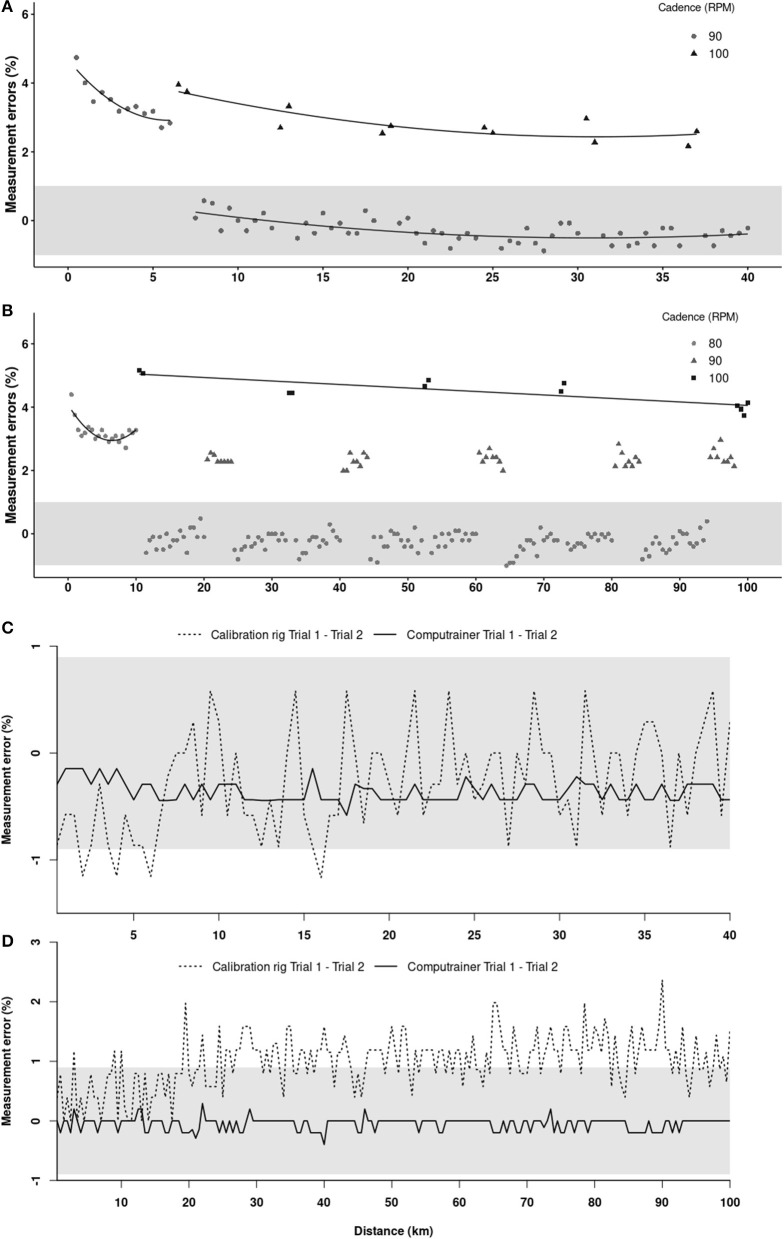
Changes in measurement errors associated with the different cadences imposed over time during the 40 **(A)** and 100 **(B)** km time-trials and between the first and second trial completed as measured by the Racermate One^™^ software and the calibration rig during the 40 **(C)** and 100 **(D)** km time-trials. The shaded areas represent the calibration rig's measurement error.

Figure 3 shows the changes in measurement errors between the calibration rig and CT associated with the different changes in cadence (or workload) over time during the 40 (A) and 100 (B) km TTs. Table 1 shows the validity-related data. In both the 40 and 100 km TTs, measurement errors during the first segment preceding the first increase to 100 RPM followed a curvilinear time-course, decreasing by ~1%, from ~4 to ~3%. Beside this initial segment, CT's measurement errors at 90 RPM for the 40 km TT and 80 RPM for the 100 km TT remained relatively constant, were within that of the calibration rig (± 0.9%) and were associated with low SEEs (~0.5%). At the highest cadence, i.e., 100 RPM, measurement errors throughout the different segments during the 40 and 100 km TTs were substantially above those of the calibration rig. Moreover, they were dependent upon the distance covered, but their fluctuations minor, as supported by SEEs ≤ 0.6%. The measurement error observed at the intermediary cadence (90 RPM) over the 100 km TT was above that of the calibration rig, albeit constant throughout the TT.

**Table 1:** Validity data.

**Distance (km)**	**Cadence (RPM)**	**Segments (km)**	**Measurement error (%)**	**Range of measurement error (%)**	**SEE or SEM (%)**	**CV (%)**
40	90–100	0–40	0.7^*^	—	± 2.3^*^	± 1.3
40	90	0.5–6	0.051x^2^−0.6x + 4.7	4.4–2.9	± 0.5	± 2.5
40	90	7.5–40	0.0014x^2^−0.085x + 0.8	0.2– −0.4	± 0.5	± 0.4
40	100	6.5–37	0.0022x^2^−0.13x + 4.5	3.7–2.7	± 0.6	± 2.1
100	80–100	0–100	0.9^*^	—	± 2.3^*^	± 1.4
100	80	0.5–10	0.027x^2^−0.35x + 4.1	3.9–3.3	± 0.4	± 2.3
100	80	11.5–94	−0.24	−0.24	± 0.4	± 0.5
100	90	20.5–98	2.4	2.4	± 0.3	± 1.7
100	100	10.5–100	−0.011x + 5.2	5.1–4.1	± 0.5	± 3.3

*CV, coefficient of variation; Measurement error, calibration rig—computrainer; RPM, rotation per minute; SEE, standard error of the estimate; SEM, standard error of measurement*;

* *not corrected for heteroscedasticity*.

### Reliability

Reliability data are reported in Table 2 and represent the differences or relationships in workload as measured by the calibration rig between trial 1 and 2 for each TTs, which is the true representation of the CT repeatability. Figure 3 shows the differences in workload measured by the CT and calibration rig during the 40 (C) and 100 (D) km TTs. It clearly highlights that when one relies on the data provided by the Racermate One^™^ software, the CT can be considered a reliable tool during both the 40 (Figure 3C) and 100 (Figure 3D) km TTs, with differences in the workloads applied to the bicycle's rear wheel between trials 1 and 2 constrained within ± 0.5%, which is below the calibration rig-associated measurement error. Another picture emerges when the calibration rig data are taken into consideration. Indeed, it can be observed that between trials 1 and 2 the differences in workloads produced by the calibration rig were higher than those of the CT for both TTs. However, the differences in workloads produced by the calibration rig between trials 1 and 2, and this for all the different segments, were within, or just slightly higher, than the measurement error of the calibration rig, as supported by the different estimated ranges of measurement errors provided in Table 2.

**Table 2:** Reliability data.

**Distance (km)**	**Cadence (RPM)**	**Segments (km)**	**Measurement error (%)**	**Range of measurement error (%)**	**SEE or SEM (%)**	**CV (%)**
40	90–100	0–40	0.3^*^	—	± 0.7^*^	± 0.4
40	90	0.5–6	−0.02x^2^ + 0.081x−0.798	−0.8– −1.0	± 0.5	± 0.5
40	90	7.5–40	−0.13	−0.13	± 0.6	± 0.4
40	100	6.5–37	−0.45	−0.45	± 0.5	± 0.2
100	80–100	0–100	1.0^*^	—	± 0.6^*^	± 0.8
100	80	0.5–10	0.0141x^2^−0.1066x + 0.5199	0.5–0.9	± 0.8	± 0.4
100	80	11.5–94	−0.0002x^2^ + 0.0266x + 0.345	0.6–1.1	± 0.8	± 0.9
100	90	20.5–98	−0.0001x^2^ + 0.0165x + 0.4741	0.8–1.1	± 0.6	± 0.7
100	100	10.5–100	−0.0002x^2^ + 0.0314x−0.0269	0.3–1.1	± 0.8	± 0.7

*CV, coefficient of variation; Measurement error, calibration rig—computrainer; RPM, rotation per minute; SEE, standard error of the estimate; SEM, standard error of measurement*;

* *not corrected for heteroscedasticity*.

## Discussion

The aim of this study was to evaluate the validity and reliability of the CT under a cadence dependent mode during variable intensity 40 km and 100 km TTs. The workloads applied to the bicycle's rear wheel by the CT were compared to those produced by a high-precision calibration rig, which has previously been used to determine the validity of the CT under a cadence-independent mode. Although cluster analyses suggest that the CT may be a valid tool to assess 40 and 100 km TT performances, close examination of findings indicates inconsistent and practically important measurement errors between the CT and calibration rig. Conversely, our results show that the CT is a reliable resistance trainer.

The average measurement error between the CT and calibration rig was < 1% for both the 40 and 100 km TTs, which is within that of the calibration rig. Such figure would indicate that the mean workload produced by the CT at the rear wheel was close to the true workload produced by the calibration rig as it is to be expected that the workload reading from the CT should always be slightly lower than that of the calibration rig because of the power lost through the bicycle's drivetrain components (Martin et al., 1998). However, important curvilinear measurement errors, some much above those of the calibration rig, were observed during the different segments of the 40 and 100 km TTs. For instance, during the first 6 km of the 40 km TT and first 10 km of the 100 km TT, measurements errors ranged respectively from 4.4% to 2.9% and 3.9 to 3.3%. Why this occurred is not clear. Variations in ambient temperature are known to affect the calibration and precision of the CT (Davison et al., 2009); however, the ambient temperature was kept constant within and between all TTs. It has been proposed that the friction of the tire against the rotating shaft of the CT creates an increase in tire temperature which reduces the force for tire deformation and therefore reduces its rolling resistance (Davison et al., 2009). To this effect, it was shown that the rolling resistance of car tires can decrease by more than 20% and take up to 20 min prior to reaching a plateau and that the effect of tire temperature on the reduction of rolling resistance was independent of the concomitant increase in tire pressure (Ejsmont et al., 2018). Although this kinetic may differ for bicycle tires, it is reasonable to believe that a similar scenario may have occurred in the current study, as a plateau in the drift emerged at the end of the 6 km segment for the 40 km TT, whereas one was already present by the end of the 10 km segment during the 100 km TT. This concords with the fact that when the 10 min warm up protocol is taken into account, about 20 min had elapsed by the end of the 6 km segment during the 40 km TT, whereas more time had accumulated by the end of the 10 km segment of the 100 km TT. It should be borne in mind that, as the intensity at which the warm-up protocol was executed is well above that recommended for an efficient warm-up in athletes (McGowan et al., 2015), this drift in measurement error during the first 10 to 20 min of a TT is likely impossible to avoid.

Following the initial segments, measurement errors between the calibration rig and CT were inconsistent throughout the TTs and apparently associated with changes in workloads. They also were dependent upon the distance covered, but this effect was rather small and insignificant from a practical point of view given that they remained within the measurement error of the calibration rig. As seen in Figure 2, every increase in workload led to an increase in measurement errors. We cannot provide a definitive explanation behind this observation. However, it is clear that the effect was systematic, with a stable reoccurrence throughout the TTs. It may potentially be related to a change in tire temperature or an issue with the algorithm used by the Racermate One^™^ software to control workload. Altogether, our results indicate that when data related to variable intensity 40 and 100 km TTs are analyzed in a single cluster, the CT will likely provide a valid estimation of the true mean power output generated by the athlete. However, the wide range in measurement errors across the different intensity segments indicates that the CT cannot provide a guarantee as to the accurateness of the power output an athlete is capable to truly maintain at any time point during exercise including various intensities.

An interesting finding of the current study is that, based on the data recorded by the Racermate One^™^ software, the CT would appear to be quite reliable, as illustrated in Figures 3C,D where measurement errors between trials 1 and 2 were within ± 0.5%. However, the differences in workloads produced by the calibration rig between the repeated trials, which provide the real representation of the CT's reliability, suggest a slightly different scenario. Indeed, as the variations in measurement errors between trials 1 and 2 were more important with the calibration rig than the CT, then it follows that the discrepancy in work produced by the calibration rig was more important than that truly measured by the CT. Under a real-life scenario this observation would suggest that despite an athlete believing that his performance is constant from one trial to the other based on the CT data, in reality he/she may have worked slightly harder or easier on one trial than the other. Our results show, however, that the difference in measurement errors between trials should not be more than ±1%. To this effect, as observed in Table 2, the range of measurement errors throughout the different segments of the 40 and 100 km TTs were constrained within the measurement error of the calibration rig. Consequently, our results indicate that the CT can reliably be used to examine differences produced by an intervention during 40 km and 100 km cycling TT performance.

This study has limitations and strengths that must be taken into account when interpreting findings. Only one CT, and 40 and 100 km TTs including 1 or 4 km bursts in power output, were evaluated. Whether the current results apply equally to all CTs or other TT type is unknown. The calibration rig made it impossible to change gears during the TTs. Hence, whether variations in measurement errors would have been similar while maintaining cadence but increasing force production through gear shifting is unknown. However, this is unlikely as the CT's algorithms are applied to the rear wheel based on wheel speed. The stochastic nature of our TTs included important variations in workloads. Had the use of TTs with smaller variations in workloads over time, which more closely mimics the pacing strategy used by athletes during real-life TTs, would have led to a better agreement between the values of the CT and the calibration rig is unclear. On the other hand, using a high-precision motor to power the bicycle instead of humans enabled removing any variability associated with biological variations and sampling error, thereby allowing precise estimation of the validity and repeatability of the CT. The TT formats used in the current study have previously been utilized by researchers and are attractive to test the effect of interventions.

## Conclusion

In conclusion, the reliability of the CT was acceptable for 40 and 100 km TTs including 1 or 4 km power bursts in power output. When data of the 40 and 100 km TTs were analyzed as a whole, the validity of the CT was also considered acceptable. However, caution must apply to this assertion in that the use of power bursts were associated with wide variations in the true power output produced by the CT, rendering impossible to determine at any one time during the TT the veracity of the workload produced by the athlete. Future studies are needed to determine the reliability and validity of the CT during less intense TTs, containing little fluctuations in intensity.

## Practical Applications

1. The CT is reliable during TTs including power bursts; hence, it will be able to detect differences > 1% between research interventions.
2. Provided that research data are analyzed as a whole to derive one single value of mean power output, the CT will likely be able to tract the validity of the mean power output produced by an athlete within 1% of the real power output generated.
3. Measurements errors of the CT can be quite variable during TTs including power bursts. Therefore, it will be difficult to determine at any one time during such a TT the legitimacy of the true workload produced by the athlete.
4. Resistance trainers using a direct-drive system, where the bicycle is linked by its chain to a cassette installed on the trainer, are obviously unaffected by issues related to variations in tire friction and temperature and, therefore, by design, may be more robust to variations in power outputs. Research assessing the validity and reliability of these resistance trainers under TT scenarios are still needed to confirm this hypothesis.

## Data Availability Statement

The raw data supporting the conclusions of this article will be made available by the authors, without undue reservation.

## Author Contributions

JG, J-MD, and EG: conceptualization. DJ and EG: formal analysis. DJ, JG, J-MD, and EG: investigation and writing—review & editing. JG, J-MD, and EG: methodology. EG and J-MD: resources and supervision. DJ: writing—original draft preparation. All authors have agreed to the published version of the manuscript.

## Conflict of Interest

The authors declare that the research was conducted in the absence of any commercial or financial relationships that could be construed as a potential conflict of interest.

## Publisher's Note

All claims expressed in this article are solely those of the authors and do not necessarily represent those of their affiliated organizations, or those of the publisher, the editors and the reviewers. Any product that may be evaluated in this article, or claim that may be made by its manufacturer, is not guaranteed or endorsed by the publisher.

## References

[B1] Adams J. D. Scott D. M. Brand N. A. Suh H.-G. Seal A. D. McDermott B. P.. (2019). Mild hypohydration impairs cycle ergometry performance in the heat: a blinded study. Scand. J. Med. Sci. Sports 29, 686–695. 10.1111/sms.13386 30659665

[B2] Amann M. Hopkins W. G. Marcora S. M. (2008). Similar sensitivity of time to exhaustion and time-trial time to changes in endurance. Med. Sci. Sports Exerc. 40, 574–578. 10.1249/MSS.0b013e31815e728f 18379223

[B3] Atkinson G. Brunskill A. (2000). Pacing strategies during a cycling time trial with simulated headwinds and tailwinds. Ergonomics 43, 1449–1460. 11083127 10.1080/001401300750003899

[B4] Berardi J. M. Noreen E. E. Lemon P. W. (2008). Recovery from a cycling time trial is enhanced with carbohydrate-protein supplementation vs. isoenergetic carbohydrate supplementation. J. Int. Soc. Sports Nutr. 5:24. 10.1186/1550-2783-5-24 19108717 PMC2626573

[B5] Cermak N. M. Gibala M. J. van Loon L. J. C. (2012). Nitrate supplementation's improvement of 10-km time-trial performance in trained cyclists. Int. J. Sport Nutr. Exerc. Metab. 22, 64–71. 10.1123/ijsnem.22.1.64 22248502

[B6] Chidnok W. Vanasant T. Hiruntrakul A. Bailey S. (2020). sEffects of high intensity interval training on peak aerobic power output and time trial performance in Thai amateur *Songklanakarin* J. Sci. Technol. 42, 1227–1232. 10.14456/sjst-psu.2020.159

[B7] Claveau P. Deshaye T. Jeker D. Pancarte T. Goulet E. D. B. (2021). Boire à sa soif vs. ad libitum: quelle différence lors d'un effort prolongé à vélo?, in Congrès de l'AQSAP (Québec).

[B8] Coakley S. L. Passfield L. (2018). Cycling performance is superior for time-to-exhaustion versus time-trial in endurance laboratory tests. J. Sports Sci. 36, 1228–1234. 10.1080/02640414.2017.1368691 28892462

[B9] Coyle E. F. Feltner M. E. Kautz S. A. Hamilton M. T. Montain S. J. Baylor A. M.. (1991). Physiological and biomechanical factors associated with elite endurance cycling performance. Med. Sci. Sports Exerc. 23, 93–107. 1997818

[B10] Davison R. C. R. Corbett J. Ansley L. (2009). Influence of temperature and protocol on the calibration of the computrainer electromagnetically-braked cycling ergometer. Int. SportMed. J. 10, 66–76.

[B11] Dionne J.-F. Lajoie C. Gendron P. Freiberger E. Trudeau F. (2018). Physiological and psychological adaptations of trained cyclists to spring cycling camps. J. Hum. Kinet. 64, 137–146. 10.1515/hukin-2017-0188 30429906 PMC6231346

[B12] Drouet J.-M. Champoux Y. Bergeron F. (2008). A user-friendly calibration system for bicycle ergometers, home trainers and bicycle power monitoring devices. Sports Eng. 11:15. 10.1007/s12283-008-0003-2

[B13] Dyer B. J. McKune A. J. (2013). Effects of music tempo on performance, psychological, and physiological variables during 20 km cycling in well-trained cyclists. Percept. Mot. Skills 117, 484–497. 10.2466/29.22.PMS.117x24z8 24611252

[B14] Ejsmont J. Taryma S. Ronowski G. Swieczko-Zurek B. (2018). Influence of temperature on the tyre rolling resistance. Int. J. Automot. Technol. 19, 45–54. 10.1007/s12239-018-0005-4

[B15] Ely M. R. Sieck D. C. Mangum J. E. Larson E. A. Brito L. C. Minson C. T.. (2019). Histamine-receptor antagonists slow 10-km cycling performance in competitive cyclists. Med. Sci. Sports Exerc. 51, 1487–1497. 10.1249/MSS.0000000000001911 30694974 PMC6579648

[B16] Evens T. Danoff J. (2019). The effects of saddle alignment and pedal stroke training on a competitive cyclist with anterior knee pain: a case report. Internet J. Allied Health Sci. Pract. 17:3.

[B17] Guiraud T. Léger L. Long A. Thébault N. Tremblay J. Passelergue P. (2010). Vo2 requirement at different displayed power outputs on five cycle ergometer models: a preliminary study. Br. J. Sports Med. 44, 449–454. 10.1136/bjsm.2007.044826 18539656

[B18] Haugen T. Pels F. Gysland T. S. Hartvigsen F. K. Høigaard R. (2020). Racing with superior and inferior team-members: an experimental test of effort changes in a cycling team sprint. Int. J. Sport Exerc. Psychol. 0, 1–13. 10.1080/1612197X.2020.1827001

[B19] Jeker D. Claveau P. Abed M. E. F. Deshaye T. Hoffman M. D. Goulet E. D. B. (2020). Drinking to thirst may not optimize prolonged cycling performance in a warm environment, in ECSS Congress (Seville).

[B20] Jeukendrup A. Saris W. H. M. Brouns F. Kester A. D. M. (1996). A new validated endurance performance test. Med. Sci. Sports Exerc. 28:266. 8775164 10.1097/00005768-199602000-00017

[B21] Jones H. S. Williams E. L. (2017). Accuracy of pacing strategy predictions in ride-alone and competitive cycling time trials. J. Sci. Cycl. 6:39–40.

[B22] Jones H. S. Williams E. L. Marchant D. Sparks S. A. Midgley A. W. Bridge C. A.. (2015). Distance-dependent association of affect with pacing strategy in cycling time trials. Med. Sci. Sports Exerc. 47, 825–832. 10.1249/MSS.0000000000000475 25121516

[B23] Lamberts R. P. (2014). Predicting cycling performance in trained to elite male and female cyclists. Int. J. Sports Physiol. Perform. 9, 610–614. 10.1123/ijspp.2013-0040a 24088710

[B24] Lamberts R. P. Swart J. Woolrich R. W. Noakes T. D. Lambert M. I. (2009). Measurement error associated with performance testing in well-trained cyclists : application to the precision of monitoring changes in training status. Int. SportMed. J. 10, 33–44. 10.10520/EJC48368

[B25] Martin J. C. Milliken D. L. Cobb J. E. McFadden K. L. Coggan A. R. (1998). Validation of a mathematical model for road cycling power. J. Appl. Biomech. 14, 276–291. 28121252 10.1123/jab.14.3.276

[B26] McGowan C. J. Pyne D. B. Thompson K. G. Rattray B. (2015). Warm-up strategies for sport and exercise: mechanisms and applications. Sports Med. 45, 1523–1546. 10.1007/s40279-015-0376-x 26400696

[B27] Micklewright D. Papadopoulou E. Swart J. Noakes T. (2010). Previous experience influences pacing during 20 km time trial cycling. Br. J. Sports Med. 44, 952–960. 10.1136/bjsm.2009.057315 19364755

[B28] Nieman D. C. Gillitt N. D. Sha W. (2018a). Identification of a select metabolite panel for measuring metabolic perturbation in response to heavy exertion. Metabolomics 14:147. 10.1007/s11306-018-1444-7 30830401

[B29] Nieman D. C. Goodman C. L. Capps C. R. Shue Z. L. Arnot R. (2018b). Influence of 2-weeks ingestion of high chlorogenic acid coffee on mood state, performance, and postexercise inflammation and oxidative stress: a randomized, placebo-controlled trial. Int. J. Sport Nutr. Exerc. Metab. 28, 55–65. 10.1123/ijsnem.2017-0198 29035597

[B30] Nieman D. C. Shanely R. A. Zwetsloot K. A. Meaney M. P. Farris G. E. (2015). Ultrasonic assessment of exercise-induced change in skeletal muscle glycogen content. BMC Sports Sci. Med. Rehabil. 7:9. 10.1186/s13102-015-0003-z 25905021 PMC4406335

[B31] Perreault-Briere M. Beliveau J. Jeker D. Deshayes T. A. Duran A. Goulet E. D. B. (2019). Effect of thirst-driven fluid intake on 1 H cycling time-trial performance in trained endurance athletes. Sports (Basel) 7:223. 10.3390/sports7100223 31615028 PMC6835292

[B32] Peveler W. W. Shew B. Johnson S. Sanders G. Kollock R. (2017). Comparison of ventilatory measures and 20 km time trial performance. Int. J. Exerc. Sci. 10, 640–648. 28674606 10.70252/JIZW9020 PMC5466407

[B33] Rønnestad B. R. Moen M. Gunnerød S. Øfsteng S. (2018). Adding vibration to high-intensity intervals increase time at high oxygen uptake in well-trained cyclists. Scand. J. Med. Sci. Sports 28, 2473–2480. 10.1111/sms.13277 30113750

[B34] Schabort E. J. Hawley J. A. Hopkins W. G. Mujika I. Noakes T. D. (1998). A new reliable laboratory test of endurance performance for road cyclists. Med. Sci. Sports Exerc. 30, 1744–1750. 9861609 10.1097/00005768-199812000-00014

[B35] Silva-Cavalcante M. D. Couto P. G. Azevedo R. de A. Gáspari A. F. Coelho D. B. Lima-Silva A. E.. (2019). Stretch-shortening cycle exercise produces acute and prolonged impairments on endurance performance: is the peripheral fatigue a single answer? Eur. J. Appl. Physiol. 119, 1479–1489. 10.1007/s00421-019-04135-4 30953177

[B36] Silva-Cavalcante M. D. Couto P. G. Azevedo„ R de A Silva R. G. Coelho D. B. Lima-Silva A. E.. (2018). Mental fatigue does not alter performance or neuromuscular fatigue development during self-paced exercise in recreationally trained cyclists. Eur. J. Appl. Physiol. 118, 2477–2487. 10.1007/s00421-018-3974-0 30155760

[B37] Sparks S. A. Williams E. Jones H. Bridge C. Marchant D. McNaughton L. (2016). Test-retest reliability of a 16.1 km time trial in trained cyclists using the CompuTrainer ergometer. J. Sci. Cycl. 5, 35–41.

[B38] van Essen M. Gibala M. J. (2006). Failure of protein to improve time trial performance when added to a sports drink. Med. Sci. Sports Exerc. 38, 1476–1483. 10.1249/01.mss.0000228958.82968.0a 16888462

[B39] Whitehead A. E. Jones H. S. Williams E. L. Rowley C. Quayle L. Marchant D.. (2018). Investigating the relationship between cognitions, pacing strategies and performance in 16.1 km cycling time trials using a think aloud protocol. Psychol. Sport Exerc. 34, 95–109. 10.1016/j.psychsport.2017.10.001

[B40] Wilkerson D. P. Hayward G. M. Bailey S. J. Vanhatalo A. Blackwell J. R. Jones A. M. (2012). Influence of acute dietary nitrate supplementation on 50 mile time trial performance in well-trained cyclists. Eur. J. Appl. Physiol. 112, 4127–4134. 10.1007/s00421-012-2397-6 22526247

